# FAMILY CAREGIVING: TIME-VARYING CONTEXTS OF SOCIAL SUPPORT, FINANCES, BURDEN, AND BENEFITS

**DOI:** 10.1093/geroni/igae098.0639

**Published:** 2024-12-31

**Authors:** Rahul Malhotra, Jeremy Lim-Soh

**Affiliations:** Chair; Co-Chair

## Abstract

Family caregiving is a crucial component of long-term care. Care provision provides opportunities for psychosocial gain but can also lead to burden among family caregivers in multiple domains – physical, psychological, social, and financial. Family caregiving is dynamic, with changes over time in the needs of care-recipients as well as care responsibilities and outcomes experienced by caregivers, yet longitudinal studies on caregiving contexts are scant. Longitudinal changes and trends in social support, financial well-being and service use, burden and benefits of caregiving, and spiritual resilience are essential to identify caregivers at-risk of detrimental changes over time. The four presentations in this symposium provide empirical findings from population-based longitudinal studies of family caregivers of older adults, most with three or more time points of data collection. The first investigates heterogeneity in perceived social support of family caregivers over time and explores the role of caregiver psychological resilience in reshaping the trajectories. The second examines longitudinal trends in financial difficulties and income among family caregivers and their impact on supportive care service utilization. The third discusses joint trajectories of the burden and benefits of caregiving over time and highlights modifiable factors in the process. The fourth unravels the paradox wherein family caregivers simultaneously experience both high burden and benefits of caregiving. Collectively, the presentations underscore the heterogeneous and dynamic nature of family caregiving and contexts over time. They also highlight the need to strengthen caregivers’ psychological resilience, financial capacity, preparedness for caregiving and spiritual beliefs to enhance their caregiving experience.

